# *Efundja* as a risk driver and change agent for the Cuvelai-Etosha basin rural communities

**DOI:** 10.4102/jamba.v16i1.1677

**Published:** 2024-10-31

**Authors:** Loide V. Shaamhula, Hendrik A.P. Smit, Justin D.S. van der Merwe

**Affiliations:** 1 School of Military Science, Faculty of Agriculture, Engineering and Natural Sciences, University of Namibia, Windhoek, Namibia; 2 Department of Geography, College of Agriculture and Environmental Sciences, University of South Africa, Pretoria, South Africa; 3 School for Geospatial Studies and Information Systems, Faculty of Military Science, Stellenbosch University, Stellenbosch, South Africa; 4 Canadian Centre for Housing Rights (CCHR), Toronto, Canada

**Keywords:** disaster risk management framework, proactive approaches, disaster risk reduction, risk driver, Sendai framework for action

## Abstract

Floods are one of the persistent major risk drivers impacting the Cuvelai-Etosha basin of northern Namibia. Locally known as *Efundja*, this disruptive event negatively impacts particularly the rural population, who have limited resources to combat its effects. Being mostly subsistence farmers in isolated communities, the floods wreak havoc with their homesteads, harvests, animals, and general way of life by cutting them off from their fields, neighbours, and essential services for prolonged periods. This study investigates the impacts and coping mechanisms of rural communities regularly affected by *Efundja*. Data was collected from four groups of respondents through interviews and focus groups. These were heads of households in the affected rural communities, the community leaders, local councillors and national government officials involved in disaster mitigation. This ensured a comprehensive picture of the impacts.

**Contribution:**

Despite the presence of a national disaster risk management strategy, the national disaster response mechanism rather reactively responds to the hazard as opposed to being proactive. Results indicates that the strategy is not fully implemented and the parts that are implemented functions as a top-down approach. Respondents reported a wide range of impacts and a general inability to effectively cope with *Efundja*, coupled with an absence of their voices in deliberations about risk reduction matters. Additions to the current disaster risk management strategy is proposed and several recommendations derived from the research results concludes the article. Should these recommendations be implemented into the Namibian disaster risk management strategy, *Efundja* as risk driver will also become an agent of change.

## Introduction

The Cuvelai-Etosha Basin^1^ is a vast transboundary wetland area in Northern Namibia, fed mainly by rivers originating in the Southern Angolan highlands (Shaamhula, Smit & Van Der Merwe 2021). Characterised by a mostly dry and semi-arid climate, the area is predicted to experience an even drier future because of climate change. Despite the unfavourable climate, the basin has a high population density because of the presence of shallow groundwater sources (locally known as *iishana*) and relatively fertile soil (Fujioka, Watanabe & Mizuochi 2018). The fertile soil and water availability attract people to settle there since they depend mainly on subsistence crop farming and animal husbandry for their livelihoods.

The main water sources of the Cuvelai-Etosha basin are the Cuvelai and Mui rivers which originate in the highlands of southern Angola. This annual influx of water, primarily from outside the basin, is locally known as *Efundja*. The name *Efundja* means ‘water that rolls in from Angola’ and is an indigenous name in the local Oshiwambo language (Shaamhula 2021). This *Efundja* water coalesces with rainwater that fell in the basin and fills up the *iishana* throughout the area (Mendelsohn, Jarvis & Robertson 2013). The effects of *Efundja* have of late become more intense as water that flows through the basin affects most of the northern area, effectively cutting off some villages and making them only accessible by air (Vatileni 2023).

During the *Efundja*, water flows through the low-lying plains, almost covering the entire surface area. This prohibits residents from accessing basic services such as health facilities, schools, markets and other amenities. The *Efundja* also damages infrastructure, homes, businesses and schools, submerging approximately 57 240 hectares of cropland annually (Vatileni 2023). As a standard response measure, the government temporarily moves affected communities to higher ground and usually accommodates them in relocation camps. In these camps, communities have restricted access to suitable accommodation, adequate sanitation facilities, potable water and items such as mosquito nets that can be used to prevent possible malaria outbreaks because of the abundance of stagnant water (Namibia Red Cross Society 2017).

Because of the recurring nature of *Efundja*, the national government got involved in assisting the impacted communities. The national government response to the persistent *Efundja* has mainly focussed on temporarily relocating the victims and providing relief aid without being proactive or addressing poverty, the root cause of what makes the people vulnerable (Shaamhula 2021).

Although these activities alleviate the immediate *Efundja* impacts, they do not address the question of what makes the people vulnerable, how to reduce the exposure to the negative effects of *Efundja*, nor help build resilience in communities as per the Namibian Disaster Risk Reduction (DRR) policy. The theory of DRR espouses DRR policies and strategies that aim to prevent different risks, reduce existing disaster risks and take care of residual risks, while at the same time strengthening resilience and the curtailment of losses as the recommended way of mitigating flood impacts (Van Niekerk 2008).

Therefore, the research reported in this article assesses *Efundja* as a main driver of risk among the rural communities of the Cuvelai-Etosha basin. The research identifies gaps and shortfalls within the national disaster risk management (NDRM) system that failed to reduce the risk in the area over the past decades. Moreover, the research highlights the lessons learned and how the existing NDRM system can be enhanced by incorporating the *Efundja* experiences of the affected communities. In doing so, experiences of how countries are dealing with disasters are enriched as well as exposing the gaps and challenges they face. This research adds value to the DRR literature through a critical analysis of the current DRR framework used in Namibia, articulating the challenges experienced by the vulnerable communities, and suggesting interventions to alleviate the current situation.

## The *Efundja* as a risk driver in the Cuvelai-Etosha basin

**FIGURE 1:**
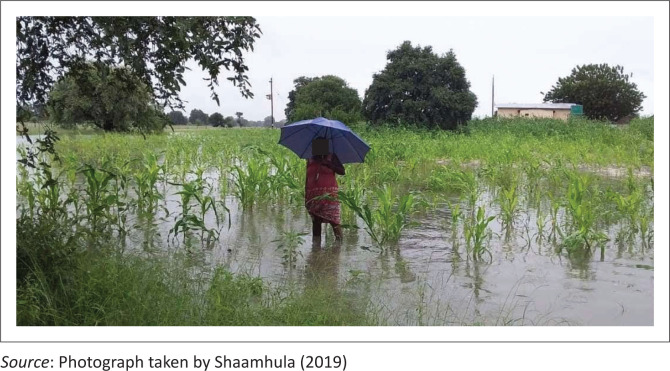
A field flooded during a severe *Efundja*.

Historically, the Cuvelai-Etosha basin received its water from the Kunene River before it diverted towards the Okavango Delta (Merron & Hocutt 2023). Currently, it is mainly fed by rainfall in the highlands of southern Angola through the Mui and Cuvelai rivers. The area where these rivers originate receives well above 900 millimetres of precipitation annually (Mendelsohn 2022). Therefore, it is primarily this water that flows through the floodplain and all the drainage lines across the basin that eventually intersect at *Omadhiya Lakes* and Etosha Pan, the lowest part of the Cuvelai basin (Mendelsohn 2022). As this water flows through the basin, it affects the entire northern area and usually completely cuts off several villages. For long periods of time, these villages can then only be reached by air, leaving them vulnerable to the effects of *Efundja* (Vatileni 2023). During this period, residents remain cut off from accessing basic services such as health services, schools, markets and other amenities. As a relief measure, communities are temporarily moved to relocation camps in elevated areas. Living in these relocation camps for extended periods can be challenging because of the lack of convenient accommodation and adequate sanitation facilities (NRCS 2017). The annual seasonal flooding in northern Namibia often causes not only damage to infrastructure but also loss of life. Figure 1 illustrates how *Efundja* usually floods fields.

Though the impact of the *Efundja* affects both urban and rural areas, rural settlements face unique challenges because their livelihoods are derived directly from the ecosystem of the basin with agricultural activities almost exclusively rainfed and climate-dependent. This study focusses on the rural inhabitants of the Cuvelai-Etosha basin and how *Efundja* acts as a major risk driver and potential agent of change in the area.

### *Efundja* in rural settlements

While both rural and urban settlements are impacted by the hazard, rural settlements are found to face unique challenges because their livelihoods are derived directly from the ecosystem of the Cuvelai-Etosha basin. This makes the rural communities especially vulnerable to the impacts of *Efundja* when compared to their urban counterparts. This observation is in line with literature, which indicates that the nature of rural settlements exposes them to unique challenges in dealing with floods as they are spread out and not as densely populated as urban settlements, making communication more difficult (Kapucu 2008). Rural communities tend to have limited resources and their rural housing structures increase their vulnerabilities. In northern Namibia, most rural housing structures are self-built individual structures. These types of structures are more easily damaged by *Efundja* when compared to urban brick-and-mortar structures that are much more permanent and stable. Furthermore, vulnerability studies revealed that rural communities that depend on small domestic markets are uniquely vulnerable and whenever the viability of these markets is impacted, livelihoods are seriously jeopardised. They emphasise that rural communities are more at risk because of their limited sources of income and constrained access to services and infrastructure (Jamshed et al. 2020). In the Cuvelai-Etosha basin, most of the rural population make a living by means of subsistence farming and use informal labour, all of which are easily disrupted by *Efundja* impacts.

### The Cuvelai-Etosha basin

**FIGURE 2:**
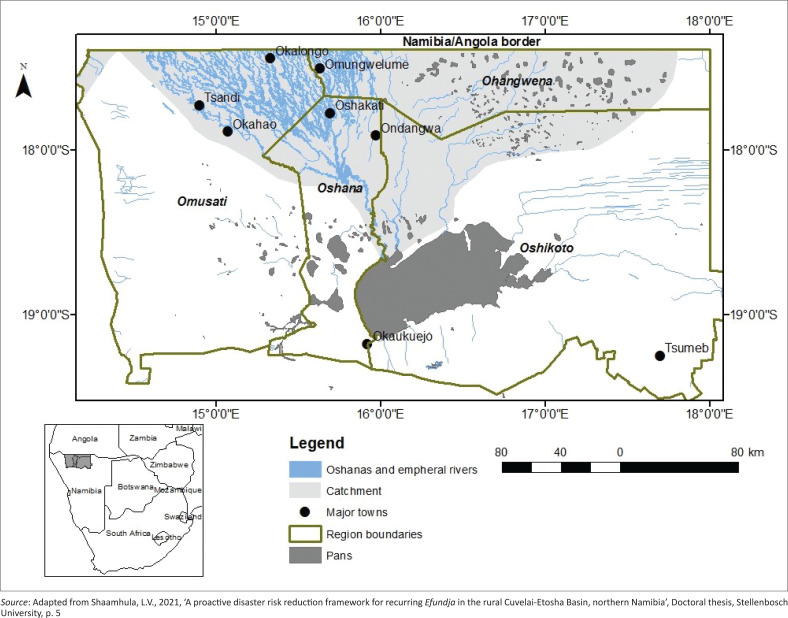
The Cuvelai-Etosha basin.

The Cuvelai-Etosha basin covers approximately 129 000 km^2^ and is a unique seasonal wetland consisting of a network of integrated waterways locally known as *iishana*, forming an ephemeral ecosystem (Figure 2) (Mendelsohn 2022).

Most of the rural residents of the Cuvelai-Etosha basin live and work in the floodplain formed by the interconnected *iishana* (Kluge et al. 2008). This basin area has attracted human settlements since the earliest of time, and the early settlers used their indigenous knowledge to construct and maintain land-use systems that were in harmony with the biophysical characteristics of the basin (Mendelsohn et al. 2013). Niipele, Kaholongo and Njunge (2015) allege that most of this knowledge has been lost during the last couple of decades and that it resulted in land management problems because residents do not know how to live in harmony with their environment anymore.

The climate of the area is characterised by low average rainfall, extreme annual temperatures, low humidity and an active wind regime, all of which enhance evapotranspiration and may contribute to the high salinity of the soils (Niipare 2020). The high evapotranspiration implies that most rainwater is not readily available to plants and this, coupled with the low rainfall, leads to a landscape characterised by aridity for most of the year (Mendelsohn et al. 2002). Although groundwater aquifers exist, only about 1% of the rainfall ends up replenishing the groundwater aquifers that many Namibians depend on. This is because of the prevailing climatic conditions and the high solar radiation experienced in the area (Midgley et al. 2005). This persistent hot and dry conditions and low, erratic rainfall have historically exposed Namibia to recurrent episodes of devastating droughts and wildfires, alternated by extreme flood events.

The *Efundja* usually takes place between March and April, damaging property and structures, and impacts the lives of people negatively. It is responsible for people displacement, loss of crops, loss of assets and disruption in the provision of services to rural communities during the rainy season on an annual basis. Although this is a historical problem, studies almost exclusively investigated its impacts, ignoring the analysis of the hazard as a driver for risk in the area or as a potential instrument for change.

This lacuna emphasises the necessity of a study of the experiences of *Efundja* to bring about change in the existing NDRM system. No previous study has analysed the approach applied by the NDRM system in dealing with the *Efundja*. The desired national approach to disasters should not be purely reactive but should concentrate on dealing with the underlying causes of the hazard. Such a more proactive approach, if applied appropriately, should alleviate the problems faced by affected rural communities, leading to positive change in the Cuvelai-Etosha basin.

### Land use in the Cuvelai-Etosha basin

Although the Cuvelai-Etosha basin contains both urban and rural settlements, the research targeted the rural villages of the Cuvelai-Etosha basin. A village in the Namibian context is a small, clustered human settlement or community located in rural areas. The housing in these villages is usually individual homes that are built out of bio-degradable materials such as grass, wood, stalks and mud (Shifidi 2015). These types of housing are more vulnerable to the effects of *Efundja* and more at risk than the mortar-and-brick housing of urban settlements since the building materials are not durable enough to withstand the prolonged impacts of moving water.

A mixed type of farming, the rearing of livestock together with crop farming, is the main land use type within the basin. This is done largely in the form of small-scale, mostly communal farming (Shifidi 2015). A typical farm consists of a household that is rearing cattle, sheep, goats, donkeys and poultry, and cultivating grain, mainly rainfed millet (*mahangu*) and sorghum (Shifidi 2015). This mixed, small-scale farming approach is the most important livelihood-securing type of activity in the area (Luetkemeier et al. 2017). The practice of agricultural subsistence farming with homesteads situated in a scattered pattern with few roads connecting them makes communication and movement difficult, especially during floods (Starkey et al. 2017).

## Global development agenda on disaster risk reduction

Since disasters had always negatively impacted mankind, they consistently responded by taking measures to mitigate the effects of such disasters. The concept of DRR emerged to address vulnerability and reduce the intensity of hazards or the exposure of people to the risks posed by such threats. To address the impacts of disasters on the population, a number of international events were held; policies and frameworks were also simultaneously developed to address the impacts of such disasters (Olowu 2010). One key framework that was established was the Hyogo Framework for Action (HFA), now replaced by the current Sendai Framework for DRR (2015–2030) which strives to reduce existing risk exposure while increasing resilience through multi-hazard assessment. This Framework aims to develop dynamic, local, preventive and adaptable systems at the global, national and local levels (Green et al. 2019; Van Niekerk, Coetzee & Nemakonde 2020). Although these frameworks are used by many states, some countries still struggle with the implementation process. A number of authors emphasised that these frameworks were influential in addressing the bigger picture, but that they were difficult to implement, because of a lack of guidelines to facilitate their implementation (Olowu 2010). The reasons behind the struggle for implementation ranged from a lack of resources to technical issues and a lack of implementation skills.

For the purpose of this article, the focus of attention is on Namibia’s endeavour to implement these frameworks effectively. Although the country has made efforts to adopt the Hyogo and Sendai goals in its disaster risk framework, the institutional structure is still rigid and does not comprehensively accommodate the development of risk reduction measures or their implementation. The country’s disaster risk management system seems to, at least theoretically, ascribe to the recommended disaster risk management approaches to disasters. However, what is practised and implemented on the ground remains reactive emergency management (Shaamhula et al. 2021).

## Research methods and design

To analyse *Efundja* as a risk driver for communities in the Cuvelai-Etosha basin, a qualitative approach was used. Focus group discussions (FGDs) and individual interviews were used to collect data on how the government currently responds to *Efundja*. The individual interview schedules and focus group schedules were developed, piloted and used to gather data from the key informants at community and national levels, providing the data for the conclusions reached by the research and reported on in this article (Shaamhula 2021). The key informant interviews (KIIs) and FGDs were combined to collect rich data while offering a variety of datasets to explain differing aspects of the research problem. Combining the methods helps identify the individual and contextual circumstances surrounding the research problem which not only adds to the interpretation of the structure of the research problem but also brings together the central characteristics of the problem across focus groups and individual interviews. This enhanced the trustworthiness of the findings. Furthermore, combining the methods helped alleviate biases arising from the use of a single observer, hence permitting data triangulation (Noble & Heale 2019).

The interview and FGD schedules went through various stages of development by eliciting responses from a panel of experts and a process of pilot testing. This section discusses the methods, profile of the respondents and the use of secondary data sources to supplement the primary data.

### Development of the instruments and their validation

The questions for the KIIs and the group interview schedules were designed by following an iterative process of analysing the available literature to generate an initial list of questions. These questions were then combined with possible questions sourced from the Namibian National Disaster Risk Management Act, Act number 10 of 2012. Open-ended, semi-structured questions were preferred to allow respondents to express themselves freely.

After the designing and structuring of interview and group discussion schedules, these instruments were reviewed by a panel of qualitative research experts during a workshop. The workshop resulted in several changes and adjustments to the instruments, culminating in interview and group discussion schedules ready for piloting.

The pilot study was conducted prior to the main data collection in the randomly selected constituency of *Etayi*, one of the identified *Efundja* affected constituencies. After incorporating the recommendations and suggestions from the pilot study, the instruments were adapted to produce the final version used to collect data during the main survey. The constituency of *Etayi*, as well as the pilot study results, were excluded from the main study.

### Purposive link: Tracing sampling

Purposive sampling with a tracing network design was used to select the study respondents. It relies on choosing respondents because of the qualities they possess (Welman, Kruger & Mitchell 2012). Therefore, respondents were purposively selected because of their knowledge and experience about *Efundja* and how it was dealt with over time. The study targeted community members, community leaders and local councillors and national disaster officials. In eliciting responses from all these role-players, the results reflect the experiences of everyone dealing with *Efundja*, from the grassroots level to the highest level of decision makers.

### Interviews

During the survey, a total of 27 KIIs were conducted, 16 with community leaders (traditional leaders), 6 with local councillors (local disaster officials) and 5 with national disaster officials. This was triangulated with FGDs conducted with community members living in the Cuvelai-Etosha basin. Focus group discussions were useful for the multiple viewpoints and responses needed on a specific topic of interest. Although the study analysed the national approach of dealing with disasters, it needed input from community members, community leaders as well as the local councillors of the affected communities. Therefore, a decision was made to use FGDs for community members and individual KIIs to survey the community leaders, local councillors and national disaster officials. The interviews generally lasted between 30 min and 1.5 h.

The national disaster officials were purposively identified, and five officials were interviewed while the local councillors were recruited based on the severity of the impacts of *Efundja* in their areas. Hence, constituencies where the impacts were severe were purposively sampled with the aid of a tracing networks design, and the researcher approached the constituency councillor’s office knowing that the constituency forms part of the sample. The councillors were interviewed and asked to identify severely impacted villages and provide the contact details of community leaders of those villages. About 27 villages were identified as the most severely impacted villages and hence these villages were visited to interview the community leaders.

In the same manner, the community leaders know each resident, particularly when they started living in the village, and therefore they were uniquely able to identify possible respondents. A prerequisite for participation was that possible respondents had to have been living in the area for at least 3 years and must have experienced *Efundja* at least three times. These criteria were designed to ensure that respondents were knowledgeable and could answer the questions in the interviews and focus group schedules. The community leaders compiled a list of residents meeting the criteria, and the researchers used it to invite possible respondents by visiting each household individually. Members of the community who indicated that they were willing to participate were then included in the study and formed part of the interview process or the FGDs.

Interview questions for local and national disaster officials assessed their role and function as disaster officials and whether they believe their respective institutions are fulfilling their respective mandates in reducing the impacts of the hazard. Interview questions for community leaders inquired about their roles and functions relating to *Efundja*. It also enquired about how they see the risk of flooding being reduced effectively and who else they think has a contribution to make in disaster risk management. Furthermore, they were asked how they prepare themselves to lead and respond to challenges, and if they see potential among community members in helping to reduce the risks posed by *Efundja*.

### Focus group discussions

Respondents in the focus group interviews ranged from 6 to 12 per group, with a total of 22 groups being interviewed. The respondents were recruited based on how long they lived in the areas and the number of *Efundja* events they have experienced. Female respondents dominated the group discussions because men go out either for work locally or to other towns, and because many households are headed by females. Questions posed in FGDs sourced information about how *Efundja* impacted them in the past in comparison to recent events, how they respond and the type of impacts they experience. Further questions solicited evidence of any preparatory measures, whether they consider permanent relocation and who they think are important actors in managing the hazard. They were also asked what they think makes communities vulnerable, how the risk of *Efundja* can be reduced, and if they know which office is responsible for managing disasters.

**FIGURE 3:**
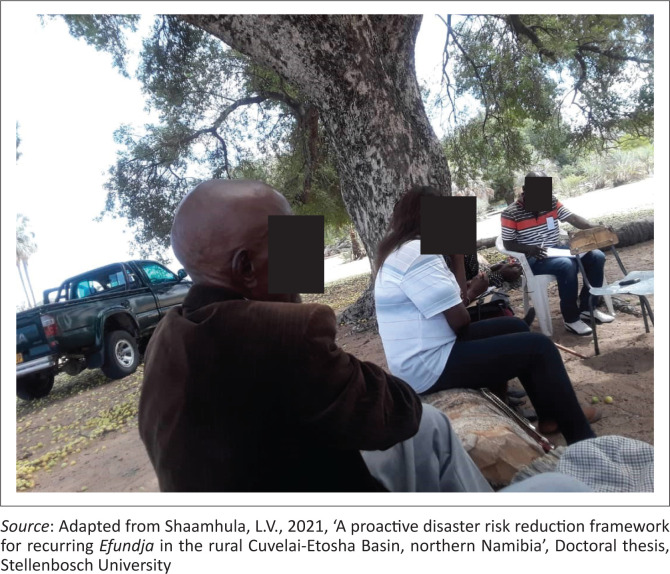
A focus group discussion in session during the survey.

After obtaining permission from the respondents, a voice recorder was used to record all group discussions and KIIs. The group discussions typically lasted about 1.5 h. However, depending on how actively respondents engaged in the discussions, some took almost 2 h to conclude. The recorded audios were transcribed, and the collected data were analysed. Figure 3 illustrates a typical setting in which the group discussions were conducted.

A total of 22 focus group interviews and 27 KIIs were conducted for this study. These data were transcribed and put together with the secondary data and loaded into ATLAS.ti (21. 21) for analysis. Through the software, the researchers created codes which were verified before grouping them into categories. These categories were used to create overarching themes which the researchers interpreted to bring meaningful information to develop the findings of the study. The software was useful in analysing data and in interpreting texts and ultimately providing a comprehensive view of the data analysed.

### Ethical considerations

All procedures performed in this study were in accordance with the ethical standards of the Stellenbosch University research committee and with the 1964 Helsinki Declaration and its later amendments or comparable ethical standards. During the process of data collection, the researchers obtained written informed consent prior to interviews, group discussions and the capturing of images and graphics. The study upheld the right to privacy through data analysis, and the respondents were allowed to withdraw at any stage during fieldwork. Ethical clearance to conduct this study was obtained from the Stellenbosch University, Social, Behavioural and Education Research (SBER) Ethics Committee (No. 10414).

## Results

This section presents the results of the survey by outlining the views of the interviewed stakeholders, namely, the community members, community leaders, local councillors and national disaster officials on how the risks associated with the *Efundja* are being dealt with. It concludes by highlighting important points of improvement needed to the existing NDRM system.

### Respondents’ length of stay in the study area

**FIGURE 4:**
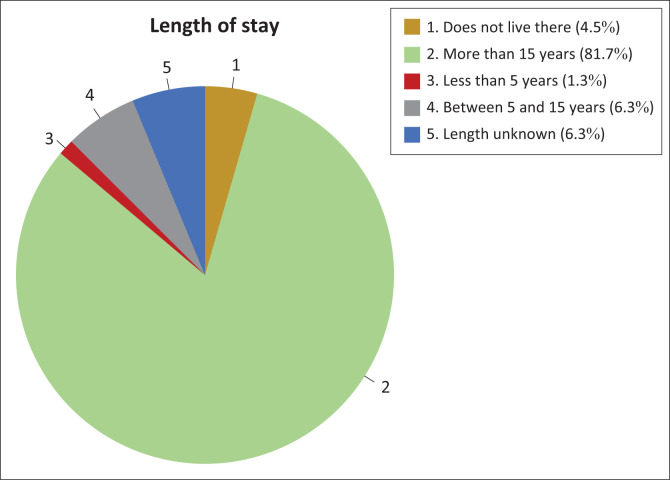
Duration of stay of respondents in the study area.

A qualifying criterion for respondents was that they should have been staying in the village for a minimum of 3 years. It was also required that respondents should have experienced *Efundja* at least three times, thereby explaining the distribution of respondent’s length of stay as depicted in Figure 4. Almost 82% of the respondents have been living in the villages in the Cuvelai-Etosha basin for more than 16 years, and most of them indicated that they have lived in their respective villages since birth.

A significant number of respondents lived in their villages for long periods of time. This implies that they were well qualified to answer questions related to *Efundja*, since they have experienced the flooding on many occasions.

### *Efundja* through the eyes of the community members

Results from FGD reveal that the occurrence of the *Efundja* is unpredictable. Respondents expressed how they would wake up to overflowing *iishana* though it did not rain. They explain how they celebrated *Efundja* in the past because it brought fish and water, which are rare resources, given the aridity of the area. In the last couple of years, it seems as if the negative consequences of the *Efundja* outweigh the positive impacts by far, and that the fish are fewer and the water less pure.

Responses from group discussions identified problems associated with *Efundja* ranging from limited access to services, an inability to move around and conduct their daily chores, and reduced access to health facilities, to a limited ability to participate in social life. All of these impacts disproportionately affect the community members because they are already a vulnerable group because of their reliance on subsistence farming and the harsh climatological conditions experienced in the basin.

One 90-year-old respondent claimed that *Efundja* changed the way the villagers lived, that it enhanced poverty, and is also changing the biodiversity of the environment in the area. Another respondent alleges:

We struggle to cook on open fire as the surface is filled with water, our small-scale business go out of business as customers became rare or completely non-existent. Our lives basically standstill. (40 year old, Female, 2020)

This quote does not only embody how *Efundja* disrupts communities’ everyday lives but also demonstrates how most livelihood securing activities grind to a standstill during the rainy season.

**FIGURE 5:**
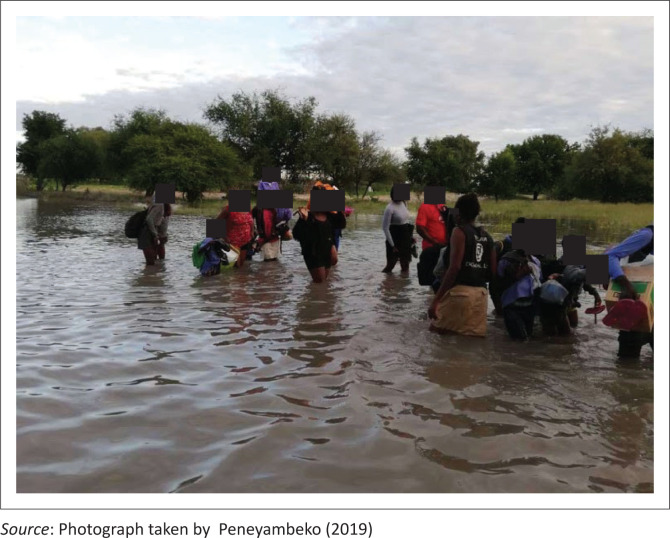
Adults assisting pupils to cross the overflowing iishana to get to school.

The communities further emphasised how *Efundja* alters the lives of their children and the way they raise their domestic animals. See Figure 5 for an illustration of one aspect of the impact.

Focus groups respondents described the process of dealing with *Efundja* to be mostly centred on local authorities working together with the national government to provide temporal shelters and relief aid. They indicated that they are aware of the procedures of reporting to the community leader through a phone call or a personal visit to receive relief aid or to be relocated. The community leader, through similar methods of communication, reports the problem to the local councillor who then visits the village and assess the level of impact. Depending on the impact, he or she writes a formal letter, or through a phone call in case of emergency, relays the information to the governor and the national government who arrange for the appropriate form of response depending on the severity of the impact.

Results from the study indicate that these impacted rural communities are living in areas that are unlike that of their urban counterparts. The communities in the Cuvelai-Etosha basin practice subsistence agriculture which prefers the area inside the basin, given the relatively arid climatic conditions. This, coupled to the lack of fresh surface water in some seasons and the fact that the land is communally owned, makes the area a destination of choice to many people. Traditionally, communities living in the basin had a way of determining what areas to use for different land use practices. However, because of the ever-increasing population, the land is becoming scarce, and priority is given to ‘owning land’, whether in flood prone areas or not. Moreover, development is also increasing, with urban commercial centres making their way into the area. While these centres promote economic development it is also believed to sometimes expand into waterways and hence increasing blockages in some of the water channels, resulting in more flooding.

Despite these conditions, people find themselves settling in these areas and remaining vulnerable to the effects of *Efundja*. In the event of *Efundja*, the results from FGDs reveal that besides the relocation camps, the national response does not include implementing steps to reduce the risk but rather focussed only on relocating the affected communities every year. A respondent declares: ‘We kept imploring the government to bring a permanent solution. We are tired of being relocated each year’. The quote evidenced both the fact that most governmental intervention is reactive in nature and that communities are dissatisfied with the way the hazard is dealt with. They recognise the futility of repeating the same procedure every time *Efundja* happens without finding proactive solutions.

Issues such as the lack of an early warning system, empowerment through education and training, poverty alleviation and the activation of community-based disaster risk management committees are further challenges affirmed by the focus groups.

Focus group discussions identified a lack of proper spatial planning. Respondents claim that people do not adhere to the spatial planning, settling in areas earmarked for other uses, or in areas where construction is prohibited. When it comes to the construction of roads and other new developments, they believe that bad execution of the spatial planning, as well as a lack of consultation, aggravates the risk of *Efundja*. During the discussions, various respondents expressed that: ‘We didn’t know there were plans to construct the road between Tsandi-Onesi. We only learn after seeing tractors clearing the area’. They stressed that they are not consulted regarding the impacts of the *Efundja.* In the case quoted above, the community believes that they could have contributed to a better solution, indicating a willingness to share their grassroots experience to find solutions to the negative impacts of the *Efundja*. It also illustrates that the communities are aware of poor spatial planning practices and that some human activities are clearly enhancing the risk, something which could have been prevented, had the community been consulted.

As far as coping mechanisms are concerned, respondents reported that they have very few effective coping strategies. During years when *Efundja* is not so severe, coping strategies include the moving of goods to higher places, constructing embankments of soil around houses, adults escorting younger children to school and increasing the height of footpaths by adding more soil. During extreme *Efundja* events, temporary relocation is the only viable option they know of.

### Community leaders and councillor’s views of the governmental response to *Efundja*

In concert with the sentiments expressed by the community members, the community leaders indicated that the only form of response they are familiar with is the relocation to the temporary relocation camps. A community leader said that: ‘We do very little in our leadership capacity. We mostly focus on coordinating the interaction between the councillors and the community members, when we should be doing more’. This quote indicates that local leaders are neither satisfied with how the impacts of *Efundja* is dealt with, nor about their current role which is mainly restricted to helping to implement temporary solutions. They added that they have been begging the government for a permanent solution, but with no response forthcoming. According to them, the governmental response to *Efundja* should be focussed on finding permanent solutions to the problems posed by the *Efundja* and not only on temporary solutions such as relocation during flooding. Local leaders also identified a lack of dedicated funding and training in disaster risk response as barriers to playing a larger role in assisting their constituency in dealing with the effects of *Efundja*.

The local councillors – who are politically appointed officials with the responsibility to oversee community affairs – are also involved in *Efundja*-related coordination at the community level. These officials indicated that their offices are under-equipped with emergency response equipment. If this could be rectified, they would be able to swiftly respond to the needs of the people during flooding. A councillor declared: ‘Our offices don’t have means of helping our people. Not even annual budgets’. They listed several items such as blankets, food products with a long shelf life, mosquito nets, first aid medical supplies, tents and heavy-duty, off-road vehicles as the most important items needed for proactively responding to *Efundja*.

In addition to equipment, they also emphasised the need for fixed budget allocations for emergency responses to *Efundja* across all councillors’ offices in the Cuvelai-Etosha basin. They indicated that the lack of financial resources accounts for their inability to effectively respond to *Efundja* and disagree with the fact that they have to rely on the central government for any and all forms of response.

The councillors explained that the decentralisation of resources would enable their offices to immediately respond to the needs of their communities. They maintained that if emergency management equipment’s are decentralised, there will be no need to wait for assistance from the central government in the capital city, which must be relayed to other offices before it reaches their offices. Another councillor stated: ‘It will bring about big change if we are given emergency equipment’s to assist our communities’. They criticised the current procedure of assistance because they see it to be a long, cumbersome procedure, which worsens the *Efundja* impacts because of the long response time. This is also evidenced by respondents stating that:

We still run when *Efundja* hit. We relocate them, give them food and go silent until the following year. This is mostly because in this country, we tend to have good policies, but they are not properly implemented. (49 year old, Male, 2020)

The local councillor’s quotes indicate dissatisfaction in the current form of response to *Efundja* and signify the need to change the response approach. However, the local councillors disagree with the notion that inadequate spatial planning aggravated the effects of *Efundja*, an allegation put forward by the community members.

Despite the challenges, these local councillors do try to address some of the identified challenges by using their own resources to purchase the required goods. Some even use their personal vehicles to deliver the necessary supplies to the affected people. Other efforts from the local councillors include imploring the regional governor’s office to avail resources to enable them to do their job.

The national DRR officials at national level seem to be mostly involved in coordination and monitoring of disaster risk response, and appear to be not well-informed about the sentiments of the communities at grassroots level. However, they acknowledged the lack of implementation of all aspects of the DRR policies and were open to better communication and coordination between communities and the DRR structures up to the highest level.

When triangulating the results from the four stakeholder groups, the different groups largely identified similar challenges. Issues such as the reactive nature of the response to *Efundja*, the type of impacts, the lack of institutional capacity and the need for more community involvement are all recognised across the groups. The only real difference was in the perception of the community that bad planning affects the severity of *Efundja*. This was denied by the councillors and government officials.

## Discussion

### The current national disaster risk reduction strategy

The Namibian government has adopted the Sendai framework as well as the former HFA (Namibia SDGs VNR Report 2021), and developed a NDRM framework for Namibia, based on the principles contained in the Sendai framework. The Namibian framework is contained in the Disaster Risk Management Act, Act number 10 of 2012, as well as in the national policy on disaster risk management. The Namibian NDRM framework stipulates the parameters within which disaster risk management should be provided in Namibia. A collaborative approach is advocated to effectively address the risks and impacts of hazards through the prevention of, preparedness for, response to and recovery from disasters. The overall vision is to be a proactive DRR framework but allows for key government decision-makers coordinating with lower levels of officials to provide swift relief aid and temporary shelter to the affected people (National Disaster Risk Management Act, Act number 10 of 2012).

While the framework was established as the guiding document on how *Efundja* impacts must be dealt with, our results show that it follows a rigid, reactive structure which does not allow enough flexibility to deal with the changing nature of *Efundja* and other hazards the country face.

Analysing the Namibian framework and assessing the situation at all stakeholder levels, reveal that there are several shortcomings that need attention before the framework can achieve the envisioned and desired DRR outcomes.

Firstly, although the NDRM framework seems to subscribe to the globally recommended disaster risk management and DRR approaches to disasters, it is evident that the framework is not fully implemented. Full implementation should be affected as a matter of urgency.

Secondly, the framework follows a top-bottom type of response, centred on key government decision-makers coordinating with other officials to provide swift relief aid and temporary shelter to the affected people. This is in contrast to the desired (and stated) collaborated approach embedded in the framework.

Thirdly, the framework does not promote the integration of all key stakeholders, namely the communities, community leaders, councillors and national disaster officials. Rather, the national disaster officials make decisions that are relayed to the communities while the affected communities wait for what is done for them without any consideration to their input.

Fourthly, the focus groups results show that some of the committees included in the NDRM framework are still non-operational.

Fifthly, our analysis reveal that the framework does not address all key vulnerability issues such as poverty and the increasing impacts of climate change that is making it difficult for people to earn livelihoods.

Due to these identified gaps in the applied NDRM framework and its implementation, this study proposes recommendations to improve and bring about change in the existing NDRM system. The following section outlines these recommendations.

## Recommendations for an improved national disaster risk management framework for Namibia

The recommended improvements to the NDRM framework are drawn up with regards to the four involved key stakeholders, namely community members, community leaders, councillors and national disaster officials.

**FIGURE 6:**
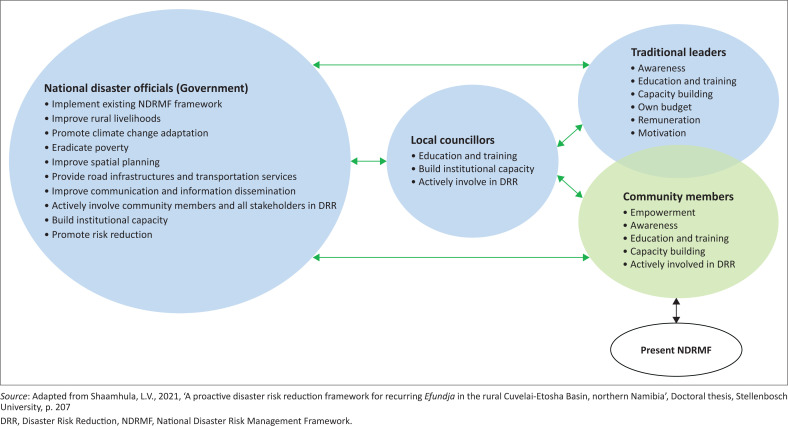
Graphical illustration of the needed improvement to the NDRMF.

Figure 6 illustrates the proposed improvement to the NDRM framework. While the authors recognise the existence of the current NDRM framework, they want to highlight its shortcomings and limitations to stimulate debate and improvements to the framework. This will ensure that the country attains its desired risk reduction goals. The recommendations emanating from this study are derived from the results gathered by the four stakeholder groups in the Cuvelai-Etosha basin: the community members, the traditional leaders, local councillors and national disaster risk officials. It particularly responds to the shortcomings in the current national DRR strategy, identified by the respondents.

As shown in Figure 6, the recommended improvements address all four stakeholder groups involved. Green arrows indicate the timely, unhindered flow of information and regular communication between the four stakeholders, the key pillars of the recommended improvements to the current situation.

In the recommended improvements, the national disaster office, the central office dealing with *Efundja*, interacts with community members (the most important role players, indicated by their position in the centre) directly or through the councillors or the community leader (this interaction is represented by a green arrow). It is important to note that this is a two-way communication and not a top-down communication experienced by communities under the present situation. This implies that the national disaster office is in continuous communication with community members by soliciting their views and opinions on any risk reduction issue.

Moreover, the national disaster office interacts with communities by supplying timeous early warning information and disseminating any other important information as soon as it becomes available. The national disaster office also empowers communities and provides training and educational opportunities to build their capacity. Empowered communities can eventually experience a radical change in behaviour and in their overall perception of *Efundja* as a hazard.

To optimally involve community members in decision-making regarding risk management, the national disaster office needs to activate community-based disaster risk management committees as stipulated by the NDRM framework. This will enable them to solicit from the community members the preparation, response and mitigation measures they employ, and incorporate that into their own disaster response programmes. This will allow the national disaster management team to develop and implement true community-based programmes and strategies, taking into account how community members deal with hazards such as *Efundja*.

In the same manner, the national disaster office should also interact with the community leaders directly or through the councillors. The community leaders should be empowered through training and empowerment opportunities as a way of building their capacity. Using these interactions, better educational and risk awareness programmes on DRR issues can be developed and presented to the community leaders. This will motivate leaders and instil confidence in their leadership roles. It will also enable them to confidently and effectively guide and direct their respective communities in times of disasters. Moreover, the national disaster office should allocate a discretionary budget to community leaders and pay them for their work and contribution to their society’s overall well-being. This will strengthen their role and may also promote better land allocation, preventing issues such as settling in flood-prone areas.

The councillors function as important links between the communities and the national disaster office. Therefore, effective, regular communication between the national disaster office and the local councillors must be maintained and expanded to ensure that the councillors are actively involved in DRR activities. By doing this, councillors can develop and lead community-based DRR programmes, bringing the government closer to the people. These councillor’s offices also require institutional capacity and empowerment to promote resilience among their respective communities. They need accessible training programmes on the concept of DRR and must be able to articulate awareness of disaster risk issues among their communities. Furthermore, these local-level disaster offices require adequate financial resources to allow them to better institute and maintain risk reduction activities.

The development, activation and management of disaster risk responses are the domain of the NDRM officials. The following recommendation is thus directed at them:

Firstly, the current NDRM framework is not implemented properly. The framework contains various risk reduction goals that, if comprehensively implemented, would enable the country to better mitigate the risk posed by disasters, such as *Efundja*.

Secondly, the livelihoods of especially rural people living in the area under study should be improved. Actions that can help alleviate poverty, such as promoting income through locally sourced, natural products, developing and investing in local markets and assisting the entrance of local people into such markets. Promoting income-generating opportunities, such as eco-tourism, to increase the welfare of the rural population and the promotion of resilient livelihoods are also options to help remedy the current situation. The national agricultural sector can also consider using agro-ecological technologies to stimulate better, more sustainable food production. Eradicating poverty in the basin and improving people’s livelihoods would help to free up resources that are currently used in welfare programmes to rather address risks and impacts of *Efundja*. It would also enable vulnerable communities to be empowered and be able to better resist disasters and face risks. If the communities are empowered and access to resources is improved, this will motivate them to work together and initiate initiatives that can help mitigate the impacts rather than feeling helpless as they do currently. Moreover, creating a synergy between social protection (improved poverty reduction measures) and DRR would promote resilience against risks.

Thirdly, climate change adaptation should be addressed as a matter of urgency by investing in strategies and policies that prepare the rural population to deal with the impacts of a changing climate. Awareness programmes should educate the population about climate change, appropriate crop diversification and the breeding of hardy types of animals, strategies that can mitigate the effects of climate change on their farming activities. Households can also consider crops that are more suitable for shorter growing seasons to improve their food security. Other coping mechanisms could be improved irrigation methods, experimenting with winter crops (because of increased winter temperatures), crop rotation, mechanisation and the sharing of farm implements. Adapting to climate change is imperative as it will enable households to improve their food security, a major challenge hampering rural populations to deal with the risks posed by *Efundja*.

Fourthly, the national response mechanism needs to improve spatial planning and enforcement for developments. Currently, it is questionable whether some of the recently constructed urban centres and roads were properly planned since they appear to block the natural water ways and hence increasing the impacts to the surrounding communities. Good spatial planning and enforcement of regulations can be accompanied by the introduction and enforcement of strict building codes and standards aimed at avoiding infrastructure developments that can hinder the free flow of water, thus aggravating the risk. Improved spatial planning will also help avoid further developments in flood-prone areas. These improvements to spatial planning include regulating land-use planning and the application of building codes and practices, improving the planning of rural road infrastructure, consultation with local communities prior to any infrastructural developments, recognising the importance of incorporating traditional knowledge in risk assessments for new developments, and developing and implementing appropriate road construction standards to ensure the natural flow of water.

Fifthly, the importance of an effective early warning system as part of the national response to disasters is of the utmost importance. Effective early warning systems are a way of enhancing preparation and ensuring the optimal use of mitigation measures. When rural communities receive timely, accurate early warning information, they can plan for the start of the rainfall season and whether the *Efundja* is expected to be severe or not. This will help people assess the severity of possible impacts, and how they should respond. Early warning information should be provided in real-time as well as interpreted in ways (including the language the warning is disseminated in) so that communities fully comprehend the message. A last proviso is that the warning should be disseminated through credible and trusted sources, and that it should reach the affected population in time for them to take action.

Sixthly, the findings reveal that some rural villages are not connected by good gravel or tarred roads. This makes it difficult for them to access services, especially during emergencies and times of *Efundja* when they can only be reached by using helicopters. Moreover, some of the existing infrastructures lack enough culverts that allow water to pass through. Hence, there is a need to provide proper road infrastructure and transportation services by connecting all villages with elevated and appropriate culvert-fitted roads to allow people access to services during the flooding season. Moreover, improving suitable transportation services in the Cuvelai-Etosha basin will enhance the mobility of the population during *Efundja*, allowing them to better cope with the impacts.

These recommendations are important because they address the underlying causes of vulnerability in the Cuvelai Etosha-basin. One of the key recommendations by the international DRR frameworks is that countries facing disasters need to address the underlying risks rather than reacting to the symptoms.

The proposed changes have the potential to revolutionise how *Efundja* is dealt with in Namibia. The knowledge gained here can then also be applied to other hazards the country may face. If used to enhance the present NDRM framework, these proposed changes will ensure empowerment and capacity building, and the use of proactive measures and social upliftment of communities; in other words, the employment of a holistic approach of dealing with disasters. Globally, this is the preferred approach to disaster risk management, something not currently catered for in the fragmented implementation of the prescripts of the current Namibian disaster risk management framework.

Moreover, the recommendations discussed in this section can be used by neighbouring countries to improve their disaster risk management frameworks, especially if they struggle with similar problems of recurring disasters, especially flooding. Countries such as South Africa and Mozambique also face recurring floods and will benefit by the incorporation of these recommendations in their policies. However, most countries of the Global South, and beyond, can adapt the recommendations to suit their unique needs and utilise them to test and improve their disaster response policies.

## Conclusion

The communities of the Cuvelai-Etosha basin face *Efundja* on an annual basis. This hazard affects almost every part of their lives ranging from impacts on their small communal farms, their businesses, their health, their cultural activities and the education of their children. Despite the impacts being severe, the overall national response to the hazard has been mainly focussed on relocation and little effort is placed on proactively reducing the risks of *Efundja*.

This article contributes to the narrative of how different countries are responding to disasters while struggling to contextualise the recommended global development agenda on risk reduction stipulated in frameworks such as the Sendai and the Hyogo frameworks of DRR. Although many choose to align themselves with these developmental agendas, they continue struggling with how to incorporate specific goals and targets into their local disaster contexts. Specifically, this article highlights how the Namibian government, which aligns itself with the Sendai framework for action, is struggling to proactively respond to the recurring *Efundja* of the Cuvelai-Etosha basin. The reasons for this inability to fully implement their own disaster risk management framework ranges from a lack of institutional capacity, lack of funding, lack of adequate implementation of existing frameworks, inadequate consultation with and empowerment of local stakeholders, the effect of climate change, to a generally vulnerable population in the Cuvelai-Etosha basin. All of these can be effectively dealt with by adopting the recommendations proposed in this study.

The findings of the research reported on in this article exposed the difficulties faced by governments and nations in applying the targets of the development agenda such as the Sendai framework for action and suggest an improved way of dealing with disasters. If the recommendation made here are properly considered and analysed by the decision-making bodies responsible for the development of these DRR frameworks, it will help future frameworks to be context-specific and promote frameworks that are comprehensive, representative and that consider the unique issues many developing countries continue to face. Moreover, the results should alert the Namibian government to the fact that it is still applying disaster management and not the intended proactive DRR it aspires to.

This study also highlights the possibility of a major risk driver, the *Efundja*, becoming a change agent. If the lessons learnt during the study are taken seriously and incorporated in the present disaster risk management framework, it can mark the onset of a new way of dealing with *Efundja* in the Cuvelai-Etosha basin. This will introduce radical changes in the basin that will ultimately lead to a better, more sustainable future for the rural communities impacted by the *Efundja*.

The lessons learned during this research process are relevant to governments, societies and groups of communities aiming to reduce and manage the risks they face. Moreover, they are relevant to the wider community on DRR and to researchers in the African context and in other areas of the Global South. Future research may include investigation of the progress involved in the implementation of the Sendai framework for action.

## Footnotes

1 This article is based on research conducted by the lead author as part of her PhD studies. The unpublished thesis is entitled: “L.V. Shaamhula. 2021. A proactive Disaster Risk Reduction Framework for recurring *Efundja* in the rural Cuvelai-Etosha Basin, northern Namibia. [Doctoral thesis]. Stellenbosch University”.
